# When mental health care is stigmatizing: experience of users and families and associated factors

**DOI:** 10.1192/j.eurpsy.2022.541

**Published:** 2022-09-01

**Authors:** K.-M. Valery, A. Prouteau, S. Guionnet, L. Violeau, T. Fournier

**Affiliations:** University of Bordeaux, Psychology, Bordeaux, France

**Keywords:** mental health professionals, schizophrénia, Families, stigmatization

## Abstract

**Introduction:**

Mental health care is considered to be one of the main sources of mental illness stigmatization. Detailed information about these stigmatization experiences is needed to reduce stigma in mental health practices.

**Objectives:**

The study aimed i) to identify the most relevant stigmatizing situations in mental health care encountered by users and families, ii) to characterize the relative importance of these situations in terms of frequency, experienced stigmatization and suffering, and iii) to identify individual and contextual factors associated with these experiences.

**Methods:**

In a focus group, users were asked to select the 15 most relevant stigmatization situations among those they elicited and those that were taken from the literature. An online survey was then conducted among users and family members to characterize these situations and identify predictors.

**Results:**

A total of 235 participants were included: 59 participants with schizophrenia diagnosis, 96 with other psychiatric diagnoses and 80 family members. The results revealed 15 situations with different levels of frequency, stigmatization and suffering. Participants with a diagnosis of schizophrenia experienced more situations of stigmatization and with a higher frequency. Moreover, factors such as recovery-oriented practices and measures without consent were the best predictors of experienced stigmatization.

**Conclusions:**

These original stigmatization situations could be targeted to reduce stigmatization and associated suffering in mental health practices. Results strongly suggest that recovery-oriented practice should be fostered to fight stigma in mental health care.

**Disclosure:**

No significant relationships.

